# A systematic review of the connection between serum uric acid levels and the risk of cardiovascular disease

**DOI:** 10.3389/fcvm.2025.1577952

**Published:** 2025-09-05

**Authors:** Jin-jin Wang, Jin-ke Yi, Li-rong Zhou, Jun Chen, Bin-qiang Zhang, Hui-min Huang, Ying Wei

**Affiliations:** ^1^ Experiment Center of Medicine, Sinopharm Dongfeng General Hospital, Hubei University of Medicine, Shiyan, Hubei, China; ^2^ Rehabilitation Department, Sinopharm Dongfeng General Hospital, Hubei University of Medicine, Shiyan, Hubei, China; ^3^ Department of Endocrinology and Metabolism, Sinopharm Dongfeng General Hospital, Hubei University of Medicine, Shiyan, Hubei, China; ^4^ Hubei Key Laboratory of Wudang Local Chinese Medicine Research, Hubei University of Medicine, Shiyan, Hubei, China

**Keywords:** serum uric acid, cardiovascular disease, risk assessment, metabolic syndrome, inflammatory response

## Abstract

Serum uric acid (SUA) has emerged as a significant biomarker for cardiovascular disease (CVD) risk assessment, garnering increasing attention in recent years. As CVD remains a leading cause of global mortality, identifying effective biomarkers for risk stratification is of paramount importance. Current evidence indicates a strong association between elevated SUA levels and increased CVD risk. However, the precise mechanisms and confounding factors underlying this relationship remain unclear. This review examines the link between SUA and CVD, exploring potential biological pathways—including metabolic syndrome, inflammatory responses, and oxidative stress—that may mediate this association. By synthesizing existing literature, this article aims to provide insights for future research and clinical applications, ultimately enhancing the understanding of SUA's utility in CVD risk evaluation.

## 1 Introduction

Serum uric acid (SUA), a product of purine metabolism, plays critical biological roles in humans. Uric acid not only acts as an antioxidant, but also exhibits Pro oxidative properties at high concentrations, which may play a key role in the development of a variety of diseases (1). The metabolism of uric acid is mainly synthesized by the liver and excreted by the kidney to maintain the balance of uric acid in the body (2). With the evolution of modern lifestyles, the increase in SUA levels has become a global phenomenon, especially under the influence of high sugar and high-fat diets, making hyperuricemia (HU) an increasingly prevalent metabolic concern (3). This trend coincides with the growing burden of cardiovascular disease (CVD), which remains the leading cause of mortality worldwide. According to World Health Organization estimates, CVD claims millions of lives annually, with its prevalence continuing to escalate (4). The risk factors of cardiovascular disease include hypertension, hyperlipidemia, diabetes, and lifestyle factors, among which the increase of SUA level is considered to be one of the potential independent risk factors (5). This study investigates the relationship between SUA levels and cardiovascular disease, focusing on the role of uric acid in the development of cardiovascular conditions and its potential clinical implications. By reviewing the relevant literature, we hope to provide a reference for clinical practice to better understand and manage SUA levels in patients with cardiovascular disease.

## 2 Search strategy

### 2.1 Search strategy

This study systematically searched the PubMed, MEDLINE, and SCIE medical databases without publication date restrictions, covering records from database inception to December 25, 2024 (last search date). We used the following key terms: “serum uric acid”, “hyperuricemia”, “cardiovascular disease”, “coronary artery disease”, “mechanism”, “risk factor”, “therapy”. Drawing on the author's experience, this narrative review selectively analyzes 45 recent and highly cited publications through a priority retrieval approach.

### 2.2 Literature exclusion criteria

1. Studies without adjusted analyses for key confounders (e.g., renal function, metabolic syndrome).
2. Non-English publications (unless critical evidence with translatable data).
3. Editorials, or conference abstracts without peer-reviewed full texts

## 3 Physiological functions and metabolism of SUA

### 3.1 Production and metabolism of uric acid

**Figure 1:**
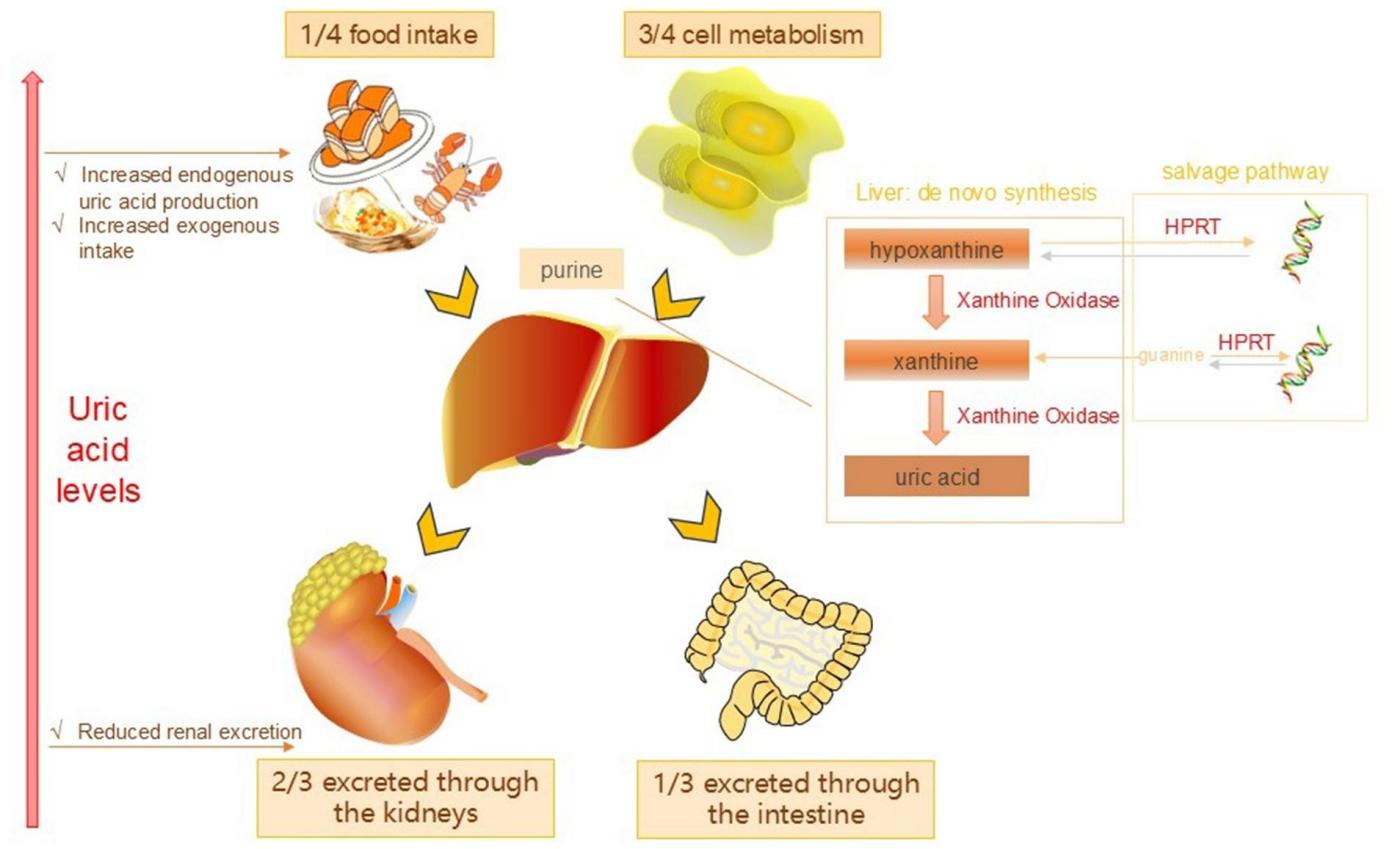
Uric acid production and metabolic pathways.

Uric acid is the final product of purine metabolism its production and excretion in the body are crucial for maintaining the balance of SUA levels. Under normal circumstances the production of uric acid mainly originates from purines in food (25%) and the metabolism of nucleic acids within the body (75%). The liver is the main organ for uric acid synthesis. Under the action of purine nucleoside phosphorylase and xanthine oxidase, purine undergoes a series of enzymatic reactions and is ultimately converted into uric acid (Figure 1). Once formed, uric acid is primarily excreted by the kidneys, with approximately 70% filtered and eliminated through urine, while the remaining 30% is expelled via the intestines (6). SUA levels are influenced by a variety of factors, such as dietary choices, genetic predispositions, kidney function and the use of specific medications. The consumption of foods high in purines can lead to an increase in uric acid production, while kidney dysfunction can hinder the body's ability to excrete uric acid, resulting in elevated levels. Certain medications, including diuretics and nonsteroidal anti-inflammatory drugs, can also influence uric acid metabolism. In addition to purine-rich foods, medications also play a significant role in the pathogenesis of HU (7). Relevant drugs can increase SUA levels by enhancing uric acid reabsorption and/or reducing uric acid secretion (Table 1). Moreover, the activity of several transporters, including URAT1 and GLUT9, is closely related to uric acid excretion, and they play an important role in uric acid reabsorption and excretion (8). Abnormal elevation of uric acid (i.e., HU) is associated with the occurrence and development of gout, hypertension, and cardiovascular diseases. Understanding the production and metabolism of uric acid is of great significance for the prevention and treatment of related diseases.

**Table 1:** Common uric acid-elevating drugs.

Drug categories	Specific drugs	Primary mechanism of action
Diuretics	Hydrochlorothiazide, Furosemide. etc	It causes an increase in SUA by reducing the excretion of uric acid by the renal tubules
Immunosuppressants	Cyclosporine, Tacrolimus	Inhibit renal excretion of uric acid and increase SUA retention
Low-dose Aspirin	Aspirin (<325 mg/day)	By inhibiting uric acid reabsorption in the kidney tubules, it reduces uric acid excretion
Antituberculosis Drugs	Pyrazinamide, Ethambutol	Pyrazinamide competitively inhibits uric acid excretion, while ethambutol reduces uric acid clearance through an unknown mechanism

### 3.2 The antioxidant effect of uric acid

Uric acid is not only the final product of purine metabolism in the body but also plays significant physiological roles, especially its antioxidant effects. Research indicates that uric acid can effectively remove free radicals in the body and reduce the damage of cells caused by oxidative stress. Uric acid can influence multiple physiological and pathological processes because of its antioxidant capacity. For instance, in conditions like chronic kidney disease and cardiovascular disease, uric acid's antioxidant effects may help slow disease progression (9). In addition, the antioxidant effect of uric acid is closely related to its concentration, and the appropriate amount of uric acid can play a protective role, but the excessive level of uric acid may lead to the increase of oxidative stress, and then lead to inflammation and tissue damage. Therefore, the concentration of uric acid in the body needs to be precisely regulated to ensure that it can play an antioxidant role without causing potential negative effects.

### 3.3 Epidemiological study on SUA and cardiovascular disease

#### 3.3.1 Epidemiological evidence: association between SUA and cardiovascular events

Epidemiological studies in recent years have revealed a significant association between SUA levels and cardiovascular disease (CVD). Multiple studies have found that high SUA levels are strongly associated with an increased risk of hypertension, coronary artery disease, heart failure, congenital heart disease, and cardiovascular death. For example, a study of 2,633 residents of a Japanese community found a U-shaped relationship between very high and very low SUA levels and cardiovascular mortality, especially in the high uric acid group, with a significantly increased risk of cardiovascular death (10). Another study noted that elevated SUA levels are strongly associated with an increased risk of chronic kidney disease (CKD) and atherosclerosis, which may be related to the effects of uric acid on endothelial function and oxidative stress (11). In addition, elevated SUA levels were found to be associated with an increased risk of coronary artery disease (CAD) in patients with type 2 diabetes, and SUA levels were positively correlated with levels of oxidation-inflammatory biomarkers and negatively correlated with serum total antioxidant capacity (TAC). This suggests that SUA can be used as a biomarker to predict CAD risk in patients with type 2 diabetes (12).

Additionally, cross-sectional studies conducted among Korean adults have shown a significant relationship between SUA levels and cardiovascular disease risk scores indicating that high uric acid levels might constitute an independent risk factor for cardiovascular disease (13). In children with dilated cardiomyopathy (DCM), it was found that increased SUA level was positively correlated with NYHA functional grade, left ventricular end-diastolic diameter, left ventricular end-systolic diameter, and left atrial diameter. It was negatively correlated with left ventricular ejection fraction and left ventricular shortening rate. Therefore, changes in SUA levels may serve as a biomarker of DCM severity in children (14). One study found that SUA levels in patients with congenital heart disease-associated pulmonary hypertension (PAH-CHD) were significantly higher than those in patients with normal pulmonary artery pressure and healthy controls, and were higher in the medium-high risk group than in the low-risk group, suggesting that SUA can be used as a useful biomarker for risk stratification and treatment response assessment in patients with PAH-CHD (15). Concurrently, investigations into congenital heart disease (CHD) pathogenesis revealed that maternal amniotic fluid metabolite analysis demonstrated significantly elevated levels of both uric acid and proline in samples associated with CHD (16). Collectively, these epidemiological studies consistently demonstrate a strong association between elevated SUA levels and increased cardiovascular event risk.

Peripheral artery disease (PAD) is one of the manifestations of systemic atherosclerosis and an important component of cardiovascular diseases. A cross-sectional study (17) of Chinese adults showed a certain association between HU and PAD. The study included 9,839 Chinese adults with hypertension, with a mean age of 63.14 ± 8.99 years, and the prevalence of HUA was 50.72%. The overall prevalence of PAD was 2.67%, with a higher rate in men (3.17%) than in women (2.25%). The proportion of HUA was also higher in men (56.82%) than in women (45.47%). After conducting multivariable logistic regression analysis separately for men and women, the study found that HUA was positively associated with PAD risk in men, with those in the highest tertile of SUA having a significantly increased likelihood of PAD. However, no such positive association was observed in women. These findings suggest that clinicians should pay attention to HU as a risk factor when assessing PAD risk in male patients.

#### 3.3.2 Large-scale cohort study: association between SUA and the risk of cardiovascular disease

Several large-scale cohort studies and randomized controlled trials (RCTS) have provided more direct evidence of a causal relationship between SUA and cardiovascular disease. The landmark Framingham Heart Study, which followed approximately 5,000 middle-aged and older participants for up to 23 years, demonstrated that elevated SUA levels were significantly associated with increased risks of multiple cardiovascular outcomes, including coronary heart disease, stroke, and heart failure (18). A prospective study showed that lowering uric acid levels could significantly improve cardiovascular outcomes in hypertensive patients. This discovery indicates that uric acid could play a significant role in both the onset and advancement of cardiovascular disease (5). While Mendelian randomization studies have not conclusively established causality between SUA and cardiovascular disease, the consistent findings from observational studies continue to support uric acid's potential pathogenic contribution (19). Therefore, there is a pressing need for additional high-quality clinical trials in the future to explore the effectiveness and mechanisms of uric acid-lowering therapy in preventing cardiovascular disease, which will ultimately provide more robust support for clinical practice.

#### 3.3.3 Differences among populations: the impact of gender, age, and race on associations

Multiple studies have investigated population-specific variations and consistently demonstrated that elevated SUA levels exert a more substantial impact on cardiovascular disease risk in women (20). Notably, while postmenopausal women may face a higher risk of cardiovascular disease in the context of HU, a study of older women in China found that high uric acid levels were significantly associated with the risk of stroke and cardiovascular disease, and showed a significant increase with age, especially in women over 50 years old (21). Chinese multi-ethnic research revealed substantial disparities in cardiovascular risk profiles between young Miao males and middle-aged Dong and Buyi females, suggesting potential interactions between age and gender factors (22). Chilunga et al. (23) s further highlighted significant variations in the hyperuricemia-cardiovascular disease association between migratory and non-migratory African populations. Their findings particularly emphasized distinct risk patterns between non-migratory rural dwellers and their urban migrant counterparts, implying potential synergistic effects of genetic predisposition and environmental influences in this relationship (23). Additionally, Yang et al. metabolic syndrome scoring system, incorporating age, sex, and racial parameters, established both a positive correlation between uric acid levels and cardiovascular risk and significant interethnic variability in this association, thereby reinforcing the critical role of racial factors in cardiovascular risk evaluation (22). Collectively, these findings indicate that the SUA-cardiovascular disease association exhibits substantial modulation by gender, age, racial background, and other demographic factors. Consequently, rigorous investigation of this relationship necessitates careful consideration of population-specific characteristics to enable accurate cardiovascular risk assessment.

### 3.4 Potential mechanism of SUA and cardiovascular disease

#### 3.4.1 Metabolic syndrome and uric acid level

Metabolic syndrome refers to a cluster of interrelated metabolic abnormalities that include insulin resistance, hypertension, hyperglycemia, and abnormal lipid metabolism, all of which together elevate the risk of developing cardiovascular disease. Studies have shown that the increase of SUA level is closely related to the occurrence of metabolic syndrome. HU frequently coexists with metabolic syndrome, demonstrating strong positive correlations with its core diagnostic components. For instance, one study found that the SUA levels in patients with metabolic syndrome were significantly higher than those in healthy controls. Within the different components of metabolic syndrome, an increase in uric acid levels was linked to an increase in waist circumference, blood pressure, and triglyceride levels (24). In addition, as an antioxidant, uric acid may be involved in the oxidative stress response related to metabolic syndrome to a certain extent, which in turn affects insulin sensitivity and secretion (25). Regulating uric acid levels may prove to be a vital approach for enhancing metabolic syndrome and its associated complications. A study conducted among the elderly Chinese population revealed a significant positive correlation between uric acid levels and metabolic syndrome, suggesting that elevated uric acid levels could serve as a potential risk factor for the development of metabolic syndrome (26). A recent study highlighted the critical role of monitoring uric acid levels in clinical environments, as it can significantly contribute to the early detection of metabolic syndrome (27).

#### 3.4.2 The association between inflammatory response and cardiovascular disease

The inflammatory response constitutes a pivotal mechanism in cardiovascular disease pathogenesis. HU is not only a risk factor for cardiovascular disease, but also may aggravate the progression of cardiovascular disease by inducing chronic inflammatory response. Research has shown that uric acid can trigger various inflammatory signaling pathways, leading to the release of pro-inflammatory cytokines such as IL-1β and TNF-α, these factors play an important role in pathological states such as atherosclerosis and heart failure (28). Uric acid plays a significant role in activating the NLRP3 inflammasome, which triggers inflammatory signaling pathways that can damage the arterial intima and contribute to plaque formation. Additionally, the buildup of uric acid may increase the risk of cardiovascular disease by affecting endothelial function, encouraging the growth of vascular smooth muscle cells, and preventing apoptosis (29). Therefore, therapies targeting HU not only reduce uric acid levels but may also improve cardiovascular health by reducing the inflammatory response.

#### 3.4.3 The role of oxidative stress in cardiovascular pathology

**Figure 2:**
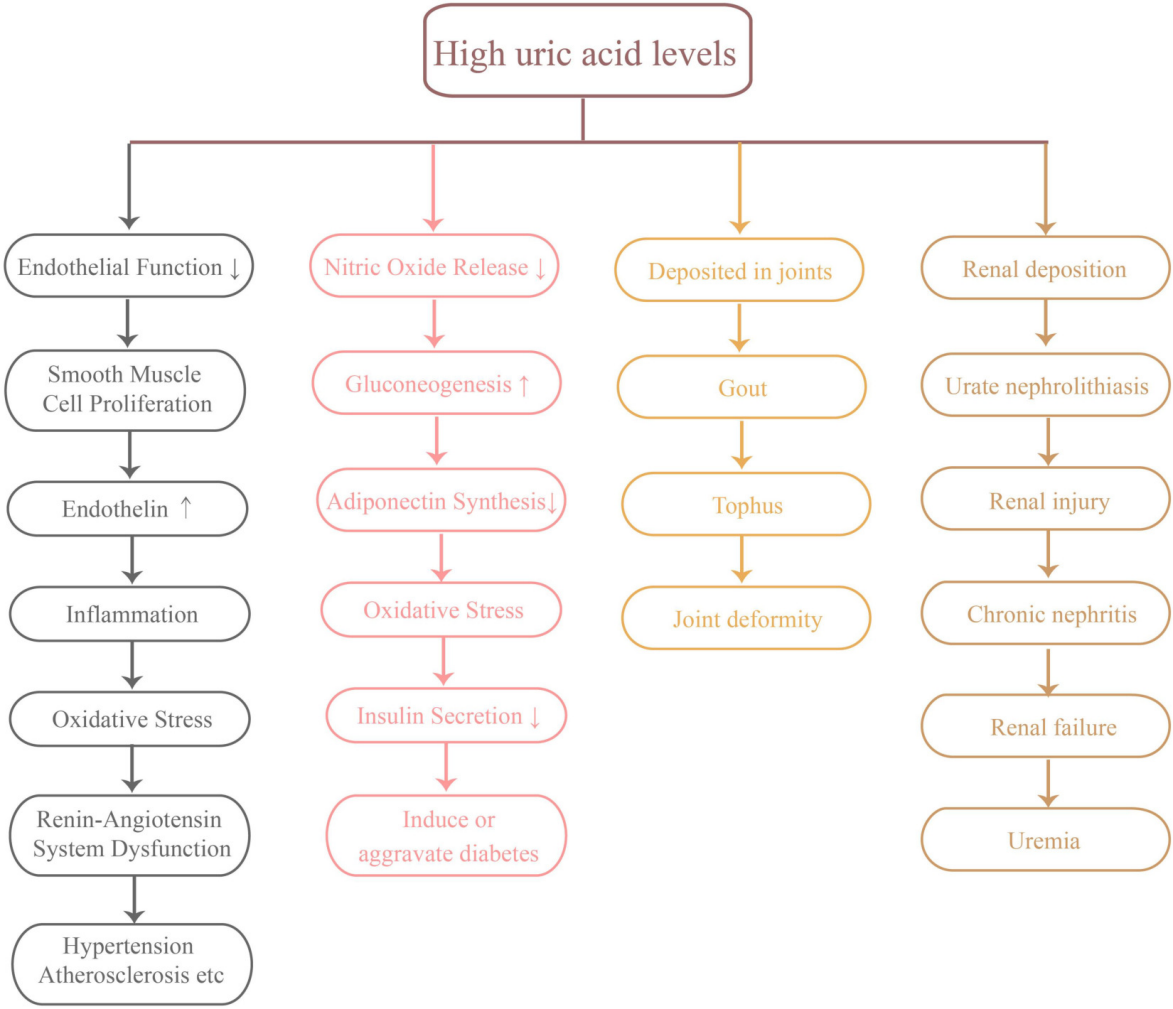
Several major hazards of high uric acid levels.

Oxidative stress refers to the imbalance between free radical generation and antioxidant defense system in the body, which plays an important role in the occurrence and development of cardiovascular diseases. Elevated uric acid levels are considered an important pathological factor in oxidative stress. Studies have shown that uric acid can worsen oxidative stress by facilitating the production of reactive oxygen species (ROS) (30). These reactive oxygen species not only directly damage cardiovascular cells, but also promote endothelial dysfunction and the formation of atherosclerosis. Additionally, oxidative stress triggers multiple signaling pathways that result in cardiomyocyte apoptosis and cardiac remodeling, which in turn accelerates the pathological progression of cardiovascular diseases (31). Reducing uric acid levels to alleviate oxidative stress could be an effective approach for preventing and treating cardiovascular diseases (Figure 2).

### 3.5 SUA levels in clinical evaluation

#### 3.5.1 Monitoring and assessment of uric acid levels

Monitoring SUA levels holds significant clinical value, particularly for evaluating chronic diseases and metabolic disorders. As the terminal product of purine metabolism, elevated uric acid concentrations may be associated with multiple pathological conditions, including gout, renal insufficiency, and cardiovascular disease. Research indicates that chronic HU can contribute to renal impairment and cardiovascular complications (32, 33). Therefore, it is essential to regularly check uric acid levels to facilitate early detection and timely intervention for these conditions. Monitoring uric acid levels can be accomplished through several methods, such as serum biochemical analysis and urinalysis. These techniques offer valuable insights into uric acid concentration and its relationship with other biomarkers, helping to assess an individual's metabolic state and potential health risks associated with elevated uric acid levels (34, 35).

The assessment of uric acid level is not limited to a single value, but also should be combined with the patient's clinical background and other biochemical indicators for comprehensive analysis. For example, uric acid levels are closely linked to renal tubular function, the inflammatory response, and metabolic syndrome (36, 37). Monitoring uric acid levels serves as a crucial reference for assessing various pathological conditions. By continuously tracking these levels, healthcare professionals can swiftly modify treatment strategies, which helps lower the risk of associated diseases and improves patient outcomes.

#### 3.5.2 Application in cardiovascular disease risk assessment

The SUA level is receiving growing attention in the assessment of cardiovascular disease risk. Substantial evidence demonstrates a significant association between elevated uric acid concentrations and cardiovascular event rates, particularly in high-risk populations including hypertensive, diabetic, and chronic kidney disease patients (38). While uric acid possesses inherent antioxidant properties that may confer some cardiovascular benefits, excessive levels paradoxically contribute to endothelial dysfunction and accelerate atherosclerotic progression (35).

Specifically, elevated uric acid levels are considered to be an independent risk factor for cardiovascular disease. Each unit increase in uric acid level is associated with an increased risk of cardiovascular disease, suggesting that clinicians should take uric acid level into account when evaluating the risk of cardiovascular disease (39). In addition, the interaction between uric acid and other cardiovascular risk factors also deserves attention. For example, the relationship between uric acid and hypertension, dyslipidemia may further aggravate the risk of cardiovascular disease (3). Detection of SUA level can not only help identify patients at high risk of cardiovascular disease, but also provide important basis for formulating individualized prevention and treatment strategies.

### 3.6 Clinical management and future research directions

#### 3.6.1 Diagnostic criteria for HU

HU is a common clinical condition characterized by elevated SUA levels. The diagnostic criteria may vary slightly depending on laboratory standards and testing methods. Generally, HU is defined as: SUA > 7 mg/dl (420 μmol/L) in men and postmenopausal women (40), SUA > 6 mg/dl (360 μmol/L) in premenopausal women (41). HU can be classified into symptomatic (gout, urolithiasis, or acute urate nephropathy) and asymptomatic forms. Studies have shown that although most asymptomatic patients do not develop urate crystal deposition-related disorders, elevated SUA can still cause tissue damage and increase the risk of hypertension, metabolic syndrome, diabetes, and cardiovascular diseases (40). Clinical recommendations (42): All gout patients should maintain SUA levels below 6 mg/dl (360 μmol/L), For severe gout cases (polyarticular involvement or tophi), SUA should be kept below 5 mg/dl (300 μmol/L).

#### 3.6.2 Effects of uric acid lowering therapy on cardiovascular disease

**Figure 3:**
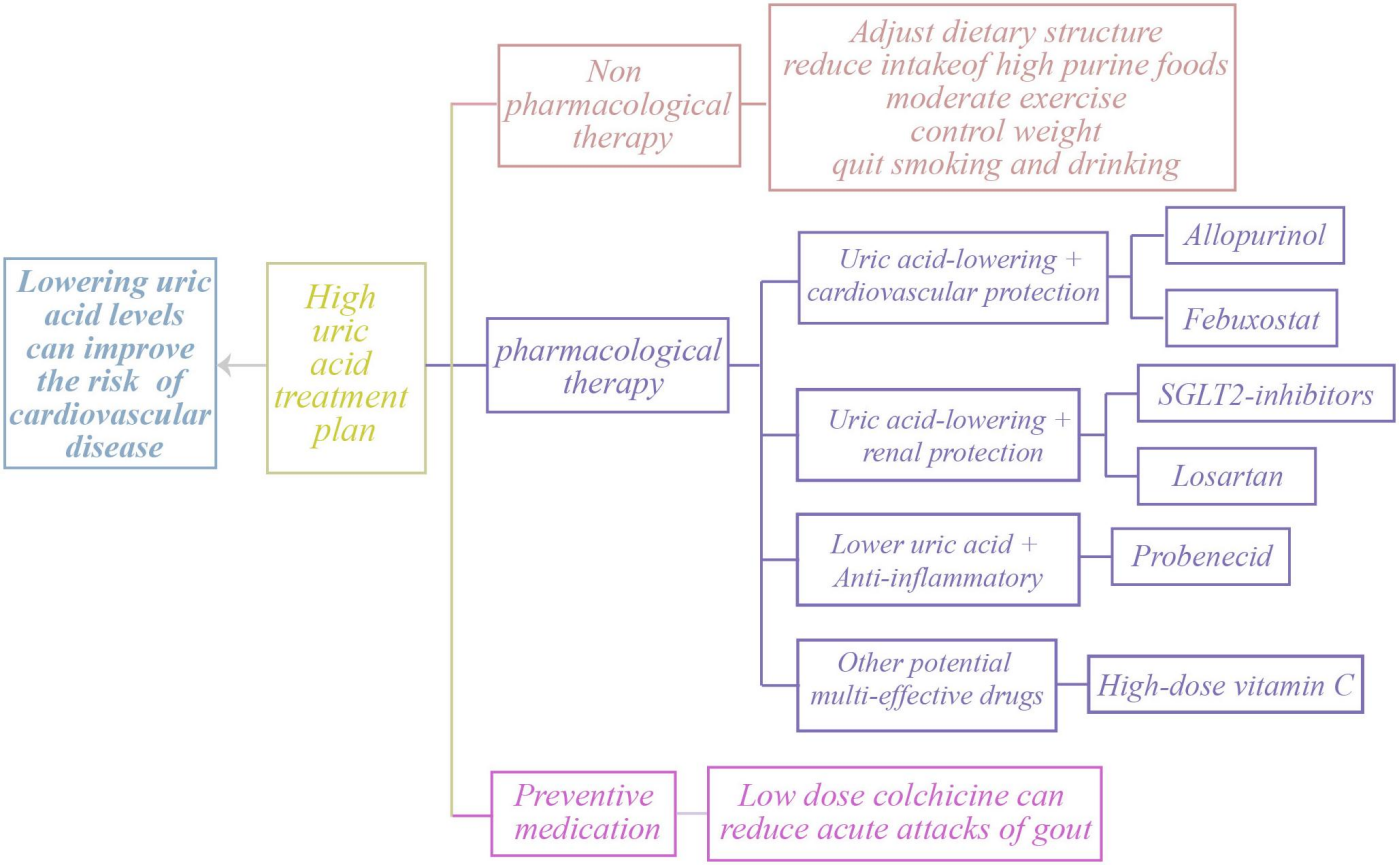
Treatment options for clinical HU.

Uric acid-lowering therapy, especially through the use of xanthine oxidase inhibitors such as allopurinol and febuxostat, has demonstrated effectiveness in lowering blood uric acid levels and has shown some improvement in cardiovascular health. Clinical trials have shown that uric acid-lowering therapy can effectively reduce both systolic and diastolic blood pressure, while also decreasing the occurrence of cardiovascular events. This evidence suggests that lowering uric acid levels may provide a protective benefit to the cardiovascular system (43). The research also found that uric acid-lowering treatment demonstrated a protective effect on the kidneys in patients with chronic kidney disease, further emphasizing the significance of uric acid control in the management of cardiovascular diseases (44). Additionally, lifestyle modifications—including dietary adjustments, weight control, reduced intake of high-purine foods, and increased consumption of fruits, vegetables, and whole grains—can lower SUA levels and consequently reduce cardiovascular risk. While current evidence supports the potential cardiovascular benefits of uric acid-lowering therapy, large-scale prospective studies remain necessary to establish its efficacy and safety across diverse populations and to determine optimal treatment strategies (Figure 3).

#### 3.6.3 Key areas and challenges for future research

Future research should prioritize several key areas to elucidate the potential and mechanisms of uric acid-lowering therapy. First, the management of asymptomatic HU remains controversial, necessitating additional randomized controlled trials to evaluate the risk-benefit profile of uric acid-lowering treatment in this population (45). Second, while uric acid-lowering medications (e.g., allopurinol and febuxostat) have demonstrated cardiovascular benefits, their long-term safety and efficacy across diverse patient groups require further investigation. Additionally, the identification and implementation of novel biomarkers could facilitate risk stratification and personalized treatment approaches (43). Finally, advancing understanding of uric acid metabolism and its cardiovascular interactions should guide development of novel therapeutics, with concurrent evaluation of their clinical safety and effectiveness. In summary, despite the promise of uric acid-lowering therapy for cardiovascular disease management, significant challenges remain that demand multidisciplinary collaboration to address.

This review examines the complex relationship between SUA and cardiovascular disease. SUA represents not merely a metabolic byproduct but also a potential contributor to cardiovascular pathogenesis. As an emerging biomarker for cardiovascular risk assessment, SUA offers clinicians novel perspectives for early detection and personalized treatment. However, academic consensus regarding the SUA-cardiovascular disease relationship remains elusive. While some studies suggest a direct causal link between HU and cardiovascular events, others posit that SUA may serve as an indirect marker influenced by concomitant metabolic disorders. These diverse research findings reflect SUA's multifaceted roles in metabolic and inflammatory pathways, underscoring the need for cautious interpretation. Future investigations should clarify SUA's precise mechanisms and clinical utility in cardiovascular diseases through large-scale prospective studies that account for population-specific biological variations, thereby enabling more targeted preventive strategies.

In conclusion, SUA's significance in cardiovascular research warrants substantial attention. Synthesizing evidence from diverse studies enables a more comprehensive understanding of this biomarker's clinical relevance and informs future directions for advancing cardiovascular prevention and treatment paradigms.
